# Decoding Three Different Preference Levels of Consumers Using Convolutional Neural Network: A Functional Near-Infrared Spectroscopy Study

**DOI:** 10.3389/fnhum.2020.597864

**Published:** 2021-01-06

**Authors:** Kunqiang Qing, Ruisen Huang, Keum-Shik Hong

**Affiliations:** ^1^School of Mechanical Engineering, Pusan National University, Busan, South Korea; ^2^Department of Cogno-Mechatronics Engineering, Pusan National University, Busan, South Korea

**Keywords:** preference levels, convolutional neural network, neuromarketing, functional near-infrared spectroscopy, commercial advertisement videos, features

## Abstract

This study decodes consumers' preference levels using a convolutional neural network (CNN) in neuromarketing. The classification accuracy in neuromarketing is a critical factor in evaluating the intentions of the consumers. Functional near-infrared spectroscopy (fNIRS) is utilized as a neuroimaging modality to measure the cerebral hemodynamic responses. In this study, a specific decoding structure, called CNN-based fNIRS-data analysis, was designed to achieve a high classification accuracy. Compared to other methods, the automated characteristics, constant training of the dataset, and learning efficiency of the proposed method are the main advantages. The experimental procedure required eight healthy participants (four female and four male) to view commercial advertisement videos of different durations (15, 30, and 60 s). The cerebral hemodynamic responses of the participants were measured. To compare the preference classification performances, CNN was utilized to extract the most common features, including the mean, peak, variance, kurtosis, and skewness. Considering three video durations, the average classification accuracies of 15, 30, and 60 s videos were 84.3, 87.9, and 86.4%, respectively. Among them, the classification accuracy of 87.9% for 30 s videos was the highest. The average classification accuracies of three preferences in females and males were 86.2 and 86.3%, respectively, showing no difference in each group. By comparing the classification performances in three different combinations (like vs. so-so, like vs. dislike, and so-so vs. dislike) between two groups, male participants were observed to have targeted preferences for commercial advertising, and the classification performance 88.4% between “like” vs. “dislike” out of three categories was the highest. Finally, pairwise classification performance are shown as follows: For female, 86.1% (like vs. so-so), 87.4% (like vs. dislike), 85.2% (so-so vs. dislike), and for male 85.7, 88.4, 85.1%, respectively.

## Introduction

The main limitation in the current commercial-video industry is that all videos are evaluated subjectively by viewing them. The objective of this paper is to develop a systematic quantitative method for evaluating consumers' preference levels when they view the videos by using a non-invasive brain image modality, functional near-infrared spectroscopy (fNIRS). In the field of recognition and classification (Moon et al., 2018; Zhang et al., 2018; Ansari et al., 2019; Kim and Choi, 2019; Kim et al., 2019; Manzanera et al., 2019; Shan et al., 2019; Yang et al., 2019; Lee et al., 2020; Leming et al., 2020; Liu et al., 2020; Lun et al., 2020; Thomas et al., 2020; Ye et al., 2020), convolutional neural networks (CNNs) have shown superior classification performance in speech detection, artificial intelligence, and multiple time-series processing compared to other conventional methods (Bengio, 2009; Kim et al., 2018). Owing to the ability of CNNs to extract essential features from acquired signals, it is used as a tool to decode the fNIRS signals. A CNN scheme suitable to extract the features from the acquired hemodynamic response signals is developed. In particular, we determined the performance of CNN-based fNIRS in decoding the input data of the hemodynamic response signals and classifying consumers' different preference levels.

A brain-computer interface (BCI), a communication bridge between the human cerebral and an external device, is utilized to detect and decode human cognition and behavior intention. BCIs are also typically used to decode the neural activity of the cerebral to restore motion function or to control machines and robots (Zander and Kothe, 2011; LaFleur et al., 2013; Degrave et al., 2019; Fiederer et al., 2019; Hu et al., 2019; Li and Shi, 2019; Furlan et al., 2020; Grossberg, 2020; Kwon et al., 2020). Recently, the application of BCI has been extended to decode consumer motivation, emotion, and decision-making (Yun et al., 2019; Giustiniani et al., 2020; Neo et al., 2020). The neural processes in consumers underlying their judgment of service-to-service brand extension are reported using different TV commercials stimulation (Yang et al., 2015; Yang and Kim, 2019). The major processes of an effective BCI system include: (a) acquisition of cerebral signals using a neuroimaging technique, (b) signal processing and analysis to obtain features representing the signal, and (c) conversion of features into commands to control devices and decode human cognition (Daly and Wolpaw, 2008; Valeriani and Poli, 2019). The BCI systems have been developed for several years based on non-invasive (Birbaumer et al., 1999; Dornhege, 2007; Pamosoaji et al., 2019) and invasive (Lal et al., 2004; Leuthardt et al., 2004) neuroimaging modalities, such as electroencephalography (EEG) (Cheng et al., 2002; Parra et al., 2002; Buttfield et al., 2006; Blankertz et al., 2007; Mellinger et al., 2007; Fazli et al., 2012; Kang et al., 2015; Park et al., 2018), magnetoencephalography (Mellinger et al., 2007; Buch et al., 2008), electrocorticography (ECoG) (Leuthardt et al., 2004), functional magnetic resonance imaging (fMRI) (LaConte, 2011; Chaudhary et al., 2017), and fNIRS (Fazli et al., 2012; Chaudhary et al., 2017; Han et al., 2018; Kang et al., 2018; Shin et al., 2018; Hong and Pham, 2019; Pham and Hong, 2020). Among these modalities, the major advantages of fNIRS are its noninvasiveness, portability, low cost, wearability, and moderate temporal and spatial resolution. Because the fNIRS is an optical modality, its acquisition types are not susceptible to electrogenic artifacts (Moghimi et al., 2012). In this study, the fNIRS was utilized as a neuroimaging modality to detect cerebral hemodynamic responses.

On the contrary, the applications of BCI were developed to improve consumer behavior cognition. The surrounding environment, including friendship and emotion, can affect product endorsement and willingness-to-pay (Liao et al., 2019). Consumer behavior, financial services, evaluation stage, and decision-making in advertising are related to neural cortex response changes in current researches, further to verify a feasibility application in neuromarketing (Senior et al., 2015; Ramsøy et al., 2018; Wei et al., 2018; Ceravolo et al., 2019; Ma et al., 2019; Hu et al., 2020). Vences et al. (2020) summarized a theoretical review of the main neural scientific research on neuromarketing's effectiveness, which is a neural measure tool, to enhance the emotional connection between consumers and organizations in social networks. Neuroscience is utilized as new access better to understand consumers' behavioral cognition, purchase decision-making, preferences feeling feedback, etc. To be specific, neuroscience was also developed to help marketers understand how to affect consumers' physiological behavior by showing some advertising and marketing strategies (Lee et al., 2007). From the researchers' perspective, the neuromarketing technique has become a novel approach to investigate commercial advertisements of different combination elements, consumer preferences, and decision-making. The neuroscience to marketing connects decoding the consumers' neurocognitive principles and the products preferred in the neuromarketing application.

The findings of Wang et al. (2016) suggest that the linear structure videos and a single brand exposure make the cortex region more active than other combinations. Identifying ways to combine various resources is a crucial decision to determine product involvement and increase preference level. The structure of an advertisement was investigated by researchers in the fields of psychology and marketing. They analyzed how the plot and script structure affect consumer behavior and gradually understood the branding product. In addition, using advertising, they attempted to increase the attention received from the audience in order to convince consumers in a better manner (Stern, 1994; Mattila, 2000; Phillips and McQuarrie, 2010). To understand the needs of consumers, marketers set goals for the desired advertising effectiveness and communication (Lavidge and Steiner, 1961; Foekens et al., 1997). Kotler (2000) summarized the process in the following three stages: (i) cognitive stage, (ii) effective stage, and (iii) behavioral stage. The level of preference toward an advertisement is considered the best measure of its effectiveness and communication. Thus, a prevalent commercial advertisement video generates a positive response toward a brand and aids it against the competition (Edith et al., 2006).

In the existing research, fNIRS has been utilized as a superior neuroimaging modality to monitor brain hemodynamic responses using neurovascular coupling compared to other techniques. Furthermore, the neurovascular coupling that captures a decrease in deoxygenated hemoglobin (HbR) and an increase in oxygenated hemoglobin (HbO) during brain activity occurs in the cerebral cortex. To conduct the experiment, multiple light emitters and detectors were employed on the fNIRS system; the wavelength of light ranged from 650 to 950 nm. The variations in the concentrations of HbO and HbR were calculated using the modified Beer-Lambert law (MBLL) (Villringer et al., 1993). Many machine learning algorithms (Naseer and Hong, 2015), such as deep learning, deep neural network, and convolutional neural network, have been applied previously in neuroscience to focus on feature extraction and improve classification accuracy. For feature extraction, the time-domain signals (Naseer and Hong, 2015) and filter coefficients from continuous and discrete wavelet transforms (DWTs) (Khoa and Nakagawa, 2008; Abibullaev and An, 2012) were shown to identify statistical properties, such as mean, skewness, kurtosis, and slope, and the measurements were based on the combined common information. In addition, for machine learning-based neuroimaging modalities, the resting-state fMRI functional connectivity-based classification has been presented using a CNN architecture (Meszlenyi et al., 2017). It also demonstrated that the application of deep learning to this research subject is suitable, given the nature of the fNIRS recordings (Rosas-Romero et al., 2019; Janani et al., 2020). Hiwa et al. (2016) analyzed brain functions by carrying out the subject classification of fNIRS data using CNN's analysis. To process signal features obtained from neuroimaging techniques, the statistical values of the time-domain signals were extracted in most previous studies. However, the size of the time window (Hong et al., 2015) and the best set of combined features (Naseer et al., 2016) are critical factors in achieving a high classification accuracy.

Overall, neuromarketing is an innovative research area to interpret consumers' competitive behaviors and decode consumers' cognition. With the development of neuroimaging tools, the fNIRS technique gradually approaches the researcher's insight to detect a brain cortex directly. Among those techniques, some basic and conventional methods, such as support vector machine, linear discriminant analysis, multiparametric linear programming, etc., are utilized to extract and classify the collected cerebral data. From previous studies in processing massive data, the conventional methods presented low intelligence, slower extraction performance, and lower classification accuracy in understanding the consumers' intention. Due to CNN's successful application, it is used in our work by demonstrating its specific structure for neuro-marketing. In a nutshell, a CNN-based fNIRS method results in a novel processing framework, which is a superior technique for feature extraction and classification.

The objectives of this paper are (i) to find out whether there exist suitable video durations for product types from the viewer's perspective (probably, there might exist an optimal duration, but only three durations were compared in this paper), (ii) to demonstrate the usage of fNIRS in accessing the consumers' intention in terms of product types and video durations, (iii) to illustrate a specific CNN structure suitable for decoding hemodynamic responses for neuro-marketing, and (iv) to develop a CNN-based decoding method for consumers' preference levels. A link between fNIRS and neuromarketing is that fNIRS is a wearable device that can measure the brain activity without asking the person' unrevealed intention: Particularly in video evaluation, an examiner with fNIRS can evaluate multiple videos at a time because fNIRS is un-harmful, noiseless, low cost, usable in an ordinary environment, etc.

The remainder of this paper is organized as follows. In section Methods and Materials, the experimental procedure, signal preprocessing and conversion, and the proposed CNN–fNIRS structures are briefly described. Sections Results, Discussion, Limitation and Future Prospects, and Conclusion present the results, discussion, limitations, and conclusions of the study.

## Methods and Materials

### Ethics Statement

The experiment was conducted upon the approval of the Pusan National University Institutional Review Board (IRB no. PNU IRB/2016_101_HR). Written consent was obtained from all subjects prior to starting, and the experimental procedure was conducted in accordance with the ethical standards stipulated in the latest Declaration of Helsinki (Santosa et al., 2013; Nguyen et al., 2016).

### Participants

In this study, eight healthy adults, comprising four females (participants 1, 2, 3, 4) and four males (participants 5, 6, 7, 8), were recruited from the Pusan National University. Table 1 shows the summarized information for eight participants (*M*_age_ = 26, *SD*_age_ = 1.85; Age_min_ = 24, Age_max_ = 29), including age, gender, and education background. In this experiment, all participants are right-hand to reduce the hemispheric-dominance difference in visual stimuli. They did not have any visual, psychiatric, or neurological disorders. Before the start of the experiment, the participants were asked to avoid drinking coffee and smoking before visual stimuli, and comprehensive instruction of the whole experimental contents was performed to all the participants. During the visual stimuli, the participants were asked to focus on each video in a relaxed position.

**Table 1:** Statistical information of participants.

**No**.	**Gender**	**Age**	**Education background**
1	Female	27	Nanomaterial Engineering
2	Female	25	Economics
3	Female	24	Mechanical Engineering
4	Female	29	Chemical Engineering
5	Male	28	Mechanical Engineering
6	Male	26	Chemical Engineering
7	Male	24	Mechanical Engineering
8	Male	25	Chemical Engineering

### Experimental Paradigm

**Figure 1:**
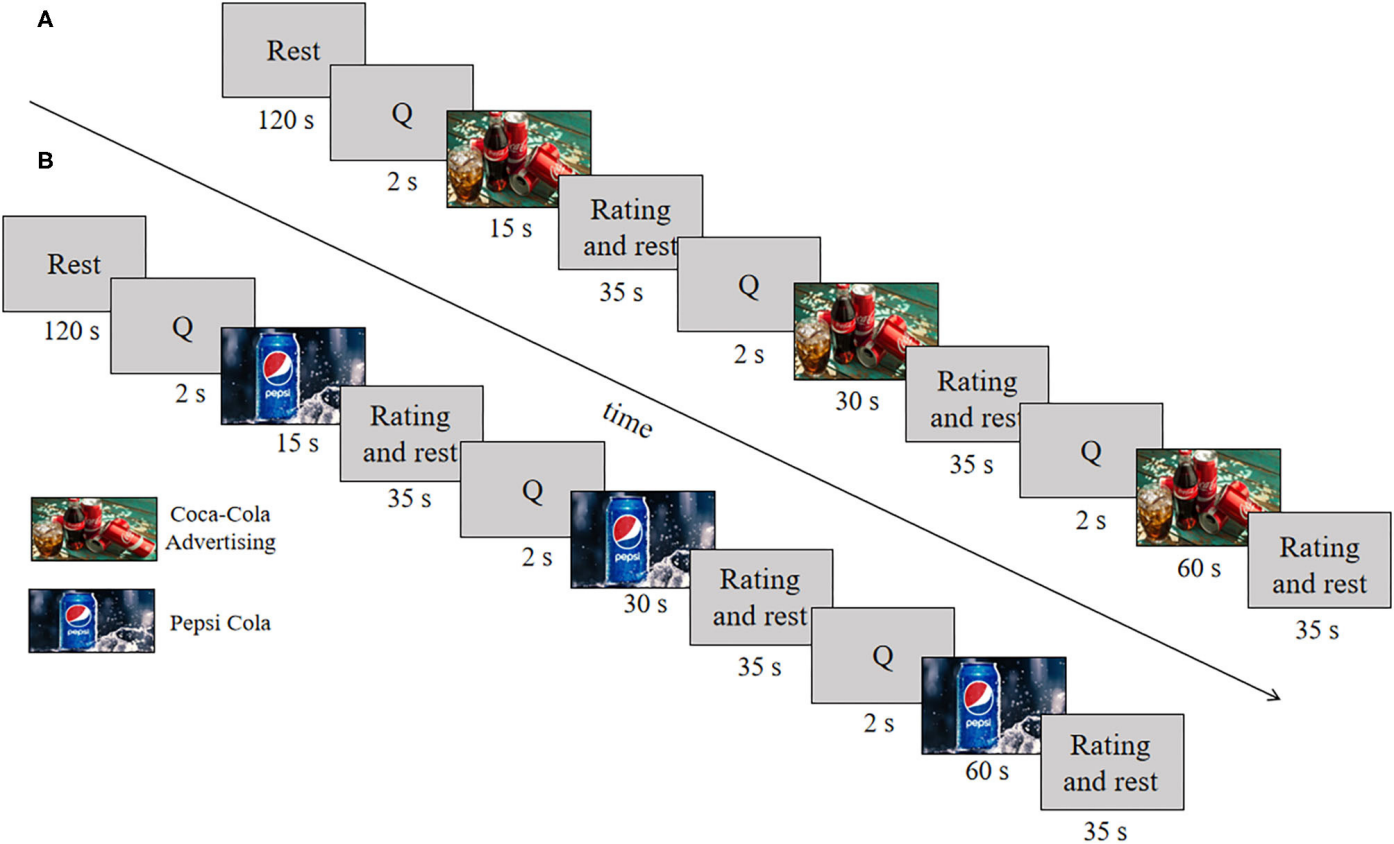
Experimental paradigm: **(A)** Coca-Cola videos, **(B)** Pepsi Cola videos.

An online survey for the brand involvement for different products was conducted to reduce the influence of a product brand during experiments and obtain appropriate commercial advertising. The videos consisted of three different brand types, including cola, chocolate, and perform advertising. Participants were asked to write a ranking score from 1 to 100 based on brand knowledge and buying behavior. According to brand involvement results, the comprehensive scores (F1: 84 ± 0.61; F2: 79 ± 0.39; F3: 80 ± 0.56) were obtained. Among those performances, the cola showed the highest score to complete the search for stimulation materials further. The commercial videos (i.e., advertising videos from Coca-Cola and Pepsi Cola) were utilized to perform the stimulation experiment: Two different types and three different durations (i.e., 15, 30, and 60 s). They were attained from the professional advertising video website using Google search. The videos with excellent resolution were shown for the first time to the participants. In a nutshell, six commercial advertising videos were divided into two types (Coca-Cola and Pepsi Cola): Each type consists of videos with three different durations (15, 30, and 60 s). In this study, a stimulation trial was followed by a rest period of 35 s (rating: 5 s, rest: 30 s). Each video was presented separately in sequence, forming three different combinations (see Figure 1).

The participants were asked to sit on a comfortable chair in front of a computer screen (Samsung LED Model: LS24A300) that displayed the experimental tasks. The viewing distance from the screen was ~45–55 cm, and the video resolution was 1,080 × 720 pixels. All commercial advertising was played on the screen in the order of duration 15, 30, 60 s. One trial consists of 2 s visual notifications, video stimulus followed by 5 s rating and 30 s rest, and the duration of video stimulus includes 15, 30, 60 s, separately. One section contains an initial 120 s rest and a 216 s task process (three trials of 15, 30, and 60 s videos were shown in sequence). The task was performed twice, resulting in a total of 12 trials. The duration of the whole experiment was 1,104 s (see Figure 1). All the participants were divided into two groups, including male group and female group. Two groups were asked to complete the experiment stimuli on the weekend, respectively.

### Behavioral Data Analysis

For the behavioral data analysis, the scores for individual trials were concluded. A statistical method called one-way analysis of variance (ANOVA) was utilized to analyze the comprehensive scores, including the video playing duration preference (Do you like this video playing duration?) and the product brand preference (Do you like this product?). The six groups of advertising in stimulating differences were statistically analyzed. Pairwise comparisons of the behavioral data were performed using Scheffe *post-hoc* tests. On the other hand, the six different commercial videos were composed of two types, including two independent variables: product branding and playing duration. An independent sample *t*-test was used to analyze the effect of the two independent variables on the preferences in the video playing duration and the product brand.

### fNIRS Data Acquisition

**Figure 2:**
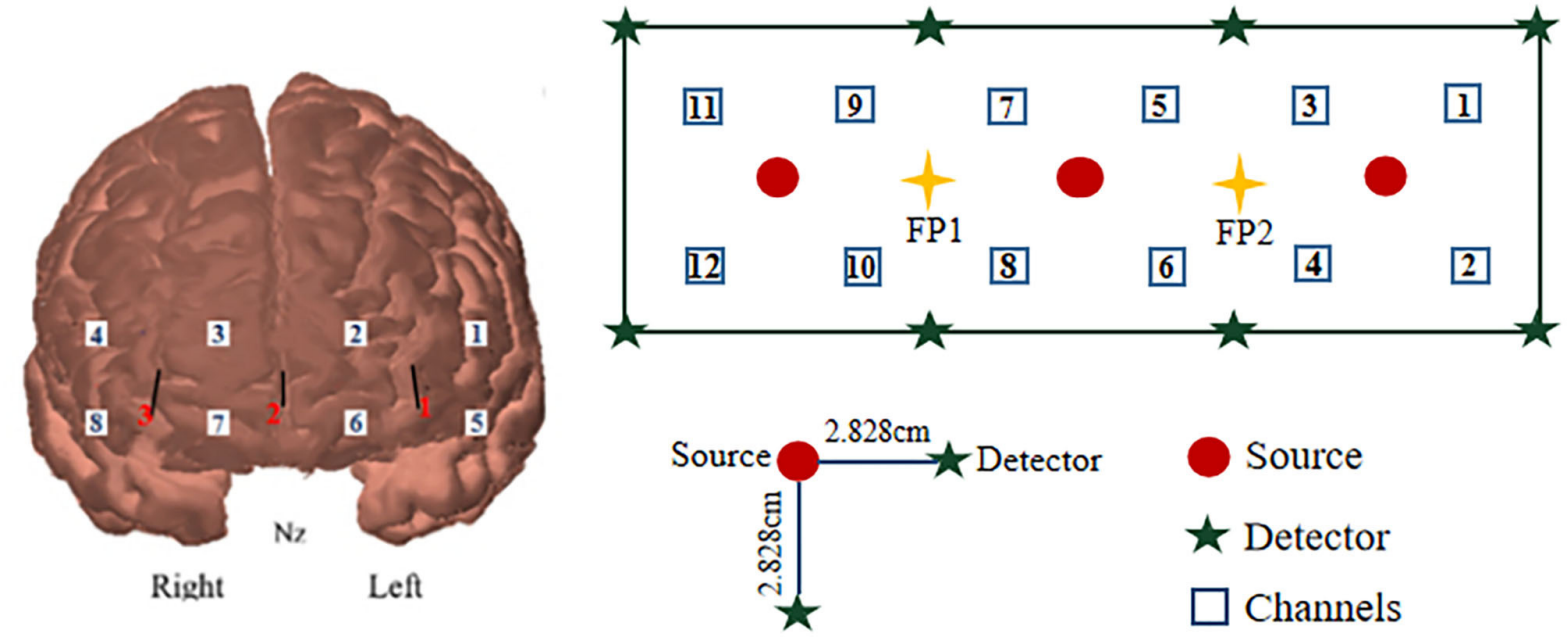
Channel configuration in the prefrontal cortex.

For the channel configuration of the cerebral prefrontal, 12 measurement channels, including three detectors and eight emitters, were placed over the prefrontal area (Figure 2). On the left and right of the prefrontal cortex, channel 1 to channel 6 and channel 7 to channel 12 were defined separately. The light has the ability to pass cortex tissue non-invasively to form the “banana” shape. Fp1 and Fp2 were utilized as the standard references for the international 10–20 system. For data acquisition, a multi-channel continuous fNIRS system (ISS Imagent, ISS Inc., USA) was utilized to measure hemodynamic responses. The system measures the optical intensities of two wavelengths (690 and 830 nm), thereby allowing the estimation of hemoglobin concentration. To acquire the signals, a sampling rate of 15.625 Hz was used, and the distance between the source and detector was 2.828 cm.

### fNIRS Data Pre-processing

The raw optical intensity data of ΔHbO and ΔHbR were obtained for all the measurement channels using the ISS Imagent data acquisition system. Next, the raw data were converted into ΔHbO and ΔHbR using the analysis software, ISS-Boxy, with the differential path factor (DPF), emitter–detector distance, and extinction coefficients of εHbO = 2.135 μM^−1^cm^−1^ and εHbO =1.791 μM^−1^cm^−1^ for the 830 nm wavelength, and εHbO = 0.95 μM^−1^cm^−1^ and εHbO = 4.93 μM^−1^cm^−1^ for the 690 nm wavelength, calculated using the modified Beer-Lambert law (MBLL) (Delpy et al., 1988).

Various physiological noises were present in the acquired hemodynamic signal, and these noises were characterized by respiration at 0.2 Hz, heart rate at 0.8 Hz, and very low-frequency oscillations at 0.03 Hz (Cui et al., 2010; Naseer and Hong, 2015). Thus, a 4th order Butterworth low-pass filter with a cutoff frequency of 0.15 Hz was utilized (Ye et al., 2009; Hong and Santosa, 2016; Zafar and Hong, 2017) to remove the physiological noises related to cardiac signals and respiration. In addition, the detrending condition was carried out inside the NIRS-SPM software to eliminate drift in the hemodynamic signal (Ye et al., 2009).

### Feature Extraction and Classification

#### Structure of CNN-Based Neuromarketing

**Figure 3:**
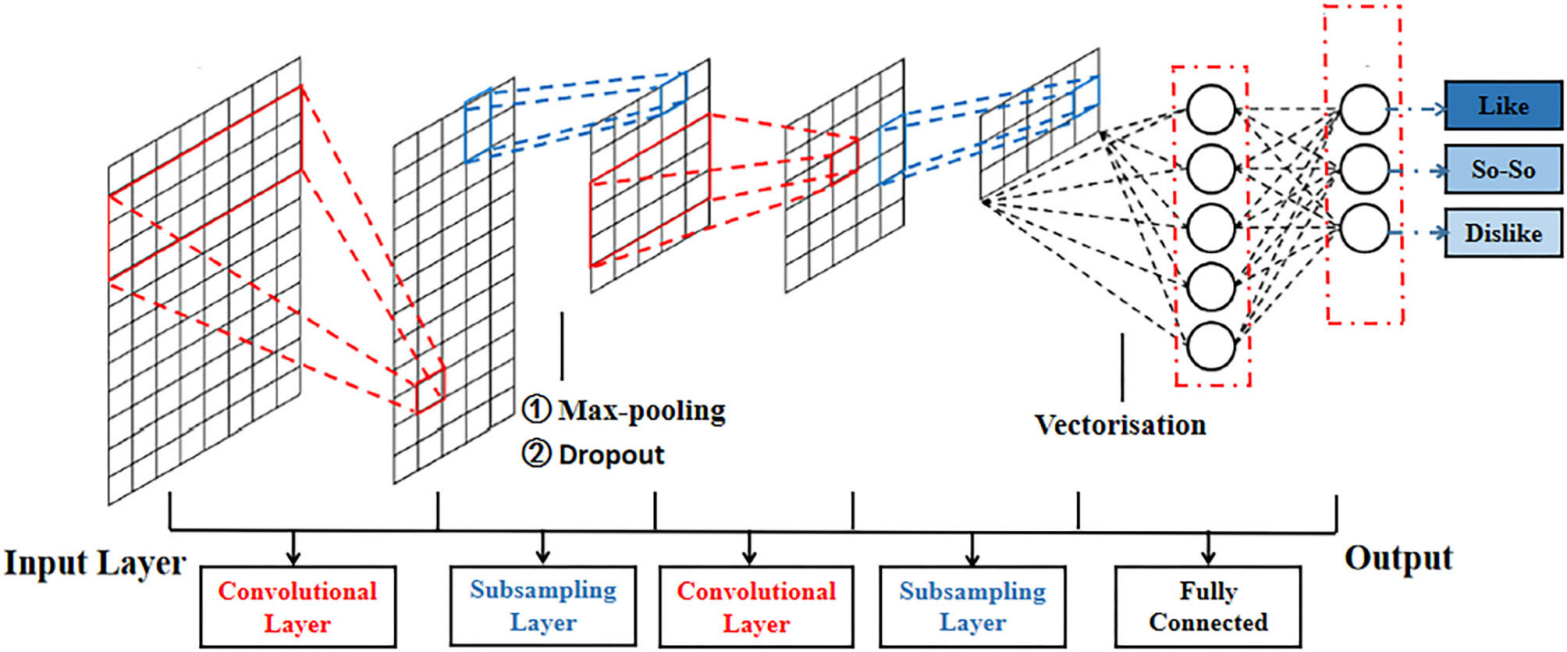
Structure of convolutional neural network for decoding consumers' preferences.

**Figure 4:**
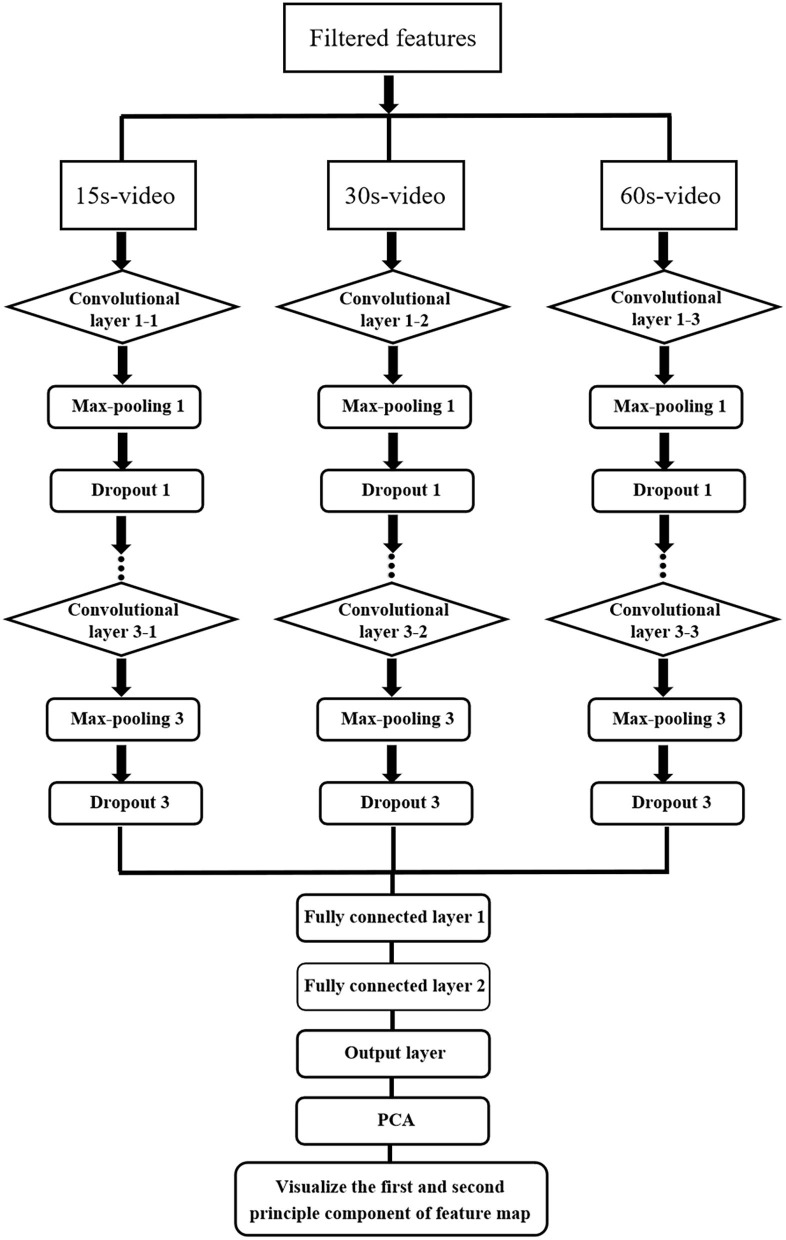
CNN decoding process.

The cognition-based evaluation of commercial advertising videos is the first step in the investigation of neuromarketing to decode consumer behavior. It is critical for neuromarketing researchers further to decode consumer behavior and preference levels in detail. Therefore, in this study, an artificial intelligence algorithm, which is a deep neural network called CNN, is presented to classify and decode different preference levels such as “dislike,” “so–so,” and “like.” In this study, according to the cerebral hemodynamic responses and the concentration of HbR, HbO changed when subjects were stimulated by commercial advertising videos, and hence, a CNN was proposed to decode these stimulation results. The structure and decoding process of CNN are presented in Figures 3, 4, respectively.

From the viewpoint of extraction and classification, a CNN that consists of several layers, including the input, convolutional, fully connected, and output layers (see Figure 3), is utilized as an automatic algorithm to train and test datasets. The width of a convolutional layer is equal to the kernel size (height) of *h*, and the dimension of the input is convolved with the input data. The output of the *i*-th filter is expressed as follows:

(1) $$\begin{array}{r} z_{i}=w\cdot x\left[i:i+h-1\right] \end{array}$$

where *w* is the weight matrix, *x*[*i*: *j*] is the submatrix of input from rows *i* to *j*, and *z* is the result value. The output layer includes three different output levels: the low- response represented by “dislike,” the mid-response denoted by “so-so,” and the high-response is “like.” With the completion of each convolutional processing, some subsampling operations, including max-pooling and dropout, are used to enhance the performance of the CNN structure. Among these operations, max-pooling is utilized as a general method to reduce data size. To avoid data overfitting, dropout is used as a regularization step to ignore one or more hidden nodes during the training process. Furthermore, the hyperparameters, such as the learning rate, batch size, and the number of epochs, are utilized to improve the classification accuracy.

**Figure 5:**
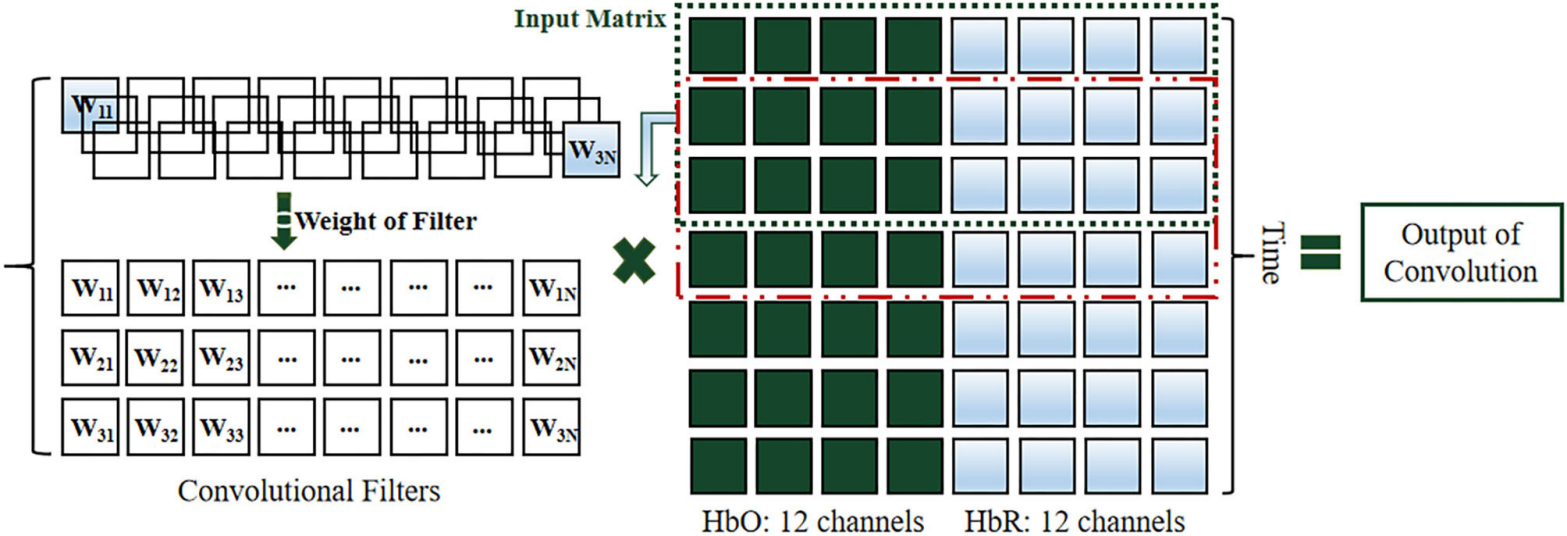
Input data: HbO (green) and HbR (light blue) of all channels. A convolutional filter was used to filter input data along the vertical axis.

The decoding process of the CNNs is shown in Figure 4. The decoding filtered features of commercial advertising consist of three subprocedures that are entered into the decoding structures of the CNN. The features of the 15, 30, and 60 s videos are utilized as input layers to build the decoding data matrix set (Figure 5). In the extraction and classification processes, the convolutional layers are processed thrice, max-pooling and dropout occur, and the fully connected operations are processed twice; all of these are critical decoding operations.

In the case of extraction and classification of each individual participant, the classifier was trained and tested using the extracted features after signal processing. Following the training step, we computed the classification accuracy using the proposed approach of the CNN-based fNIRS. In the subsequent section, the details of the proposed CNN structure and hemodynamic conversion are discussed.

#### Proposed Structures of Convolutional Neural Networks

This paper presents a novel investigation to identify behavior cognition. The proposed CNN structure was used to decode the different preference levels of consumers. As an automatic extractor and classifier, the structure can achieve a high classification performance. For the processing of input data, Figure 5 shows a conversion method to present the changes in the concentrations of HbO and HbR in all prefrontal channels, and the overall process was presented using the dataset matrix to replace the common image processing of the CNN structure. The M by N matrix represents the input data of the CNN, where M denotes the number of points during a period based on the sampling rate (M = time × sampling rate), and the period a is set according to the duration of videos (15, 30, and 60 s). The numbers of channels for HbO and HbR (12 channels each) are represented by N. In addition, three structures of the CNN are considered: CNN with one convolutional layer (CNN21), two convolutional layers (CNN2), and three convolutional layers (CNN3). Furthermore, Table 2 presents the numbers of filters for each CNN structure.

**Table 2:** Number of filters for each CNN structure.

**Structure types**	**Convolutional layer**	**Filters in each convolutional layer**
CNN 1-a	1	8
CNN 1-b	1	16
CNN 2-a	2	8, 8
CNN 2-b	2	16, 16
**CNN 3-a**	**3**	**8, 8, 8**
CNN 3-b	3	16, 16, 16

*The bold values indicate the proposed method in this paper*.

The processing of input data along the vertical axis involved a one-dimensional convolution (Figure 5). The crucial elements of the convolution consisted of convolutional filters in the convolutional layers and the input dataset matrix of cerebral hemodynamic conversion. To conduct data training during the convolution process, a typical algorithm (He, 2016) was used to automatically update the weight values of filters of each convolutional layer, and the kernel size of the filters was 3. After each convolutional layer, max-pooling with a kernel size of 2 was utilized to search for more useful data, followed by the dropout step with a dropout rate of 50%. The first and second connected layers based on the output layer contained 52 and 26 hidden nodes, respectively. The output layer had three nodes corresponding to three cases, which presented high-activation, mid-activation, and low-activation. They were classified using the softmax function. To better understand the CNN structure used in this study, Table 3 presents the input and output sizes of each layer in our proposed CNN3-a.

**Table 3:** Input and output sizes of CNN 3-a for 15 s video.

**Layer**	**Input size**	**Output size**	**Properties**
Convolutional layer 1	208, 24	208, 12	8 filters with kernel size 3
Max-poling 1	208, 12	104, 12	Kernel size 2
Dropout 1	104, 12	104, 12	Dropout rate 50%
Convolutional layer 2	104, 12	104, 12	8 filters with kernel size 3
Max-poling 2	104, 12	52, 12	Kernel size 2
Dropout 2	52, 12	52, 12	Dropout rate 50%
Convolutional layer 3	52, 12	52, 12	8 filters with kernel size 3
Max-poling 3	52, 12	26, 12	Kernel size 2
Dropout 3	26, 12	26, 12	Dropout rate 50%
Fully connected layer 1	312	52	52 hidden layers
Fully connected layer 2	52	26	26 hidden layers
Output layer	26	3	3 hidden layers

For the proposed structure, a rectified linear unit (ReLU), which is a nonlinear function, was utilized to activate all layers in the CNN structures, as shown in Nair and Hinton (2010). Compared to other

(2) $$\begin{array}{r} \alpha \left(x\right)=\{\begin{array}{cc} 0, & x<0\\ x, & x\geq 0 \end{array} \end{array}$$

activation functions, the ReLU function can improve the training process of deep neural network architectures for complex and large-scale data sets, avoid a vanishing gradient, and in practice, achieve a much faster convergence to the optimum point. Furthermore, the hyperparameters of the CNN, such as learning rate, number of epochs, and batch size, were utilized to train all the CNN structures. These parameters were selected for each individual participant using the grid search method (Table 4). Adam was applied as a gradient descent optimization algorithm, whose parameters β_1_, β_2_, and ε were set as 0.9, 0.1, and 10^−8^, respectively (Kingma and Ba, 2015).

**Table 4:** Hyperparameters of each individual subject for CNN.

**CNN**	**Subject 1**	**Subject 2**	**Subject 3**	**Subject 4**	**Subject 5**	**Subject 6**	**Subject 7**	**Subject 8**
Epochs	100	100	100	100	100	100	100	100
Batch size	8	24	16	8	24	16	8	8
Learning rate	0.0005	0.001	0.0005	0.0005	0.001	0.001	0.0005	0.0005

#### Convolutional Filters of Decoding Framework

One insight of CNN can distinguish three different preferences for each duration video by updating its filters' weight values in this work. Thus, to ensure the performance of CNN's filters, identifying the distinguishable channel input is a crucial operation to examine the first layer of CNN. The forward and backward propagation are utilized for training the collected data. CNN can learn how to emphasize some channels containing distinguishable signals with increasing the related weight values because of the interaction between each column of filters. Each channel achieved input data. After data training, each convolutional filter's column was averaged to approach the most distinguishable channel. Finally, the channel for all the input data samples with the highest weight value of an averaged convolutional filter was concluded for visualization. In a nutshell, each convolutional filter has a specific task to identify the preference levels. For each specific video duration, its convolutional filter of decoding CNN framework is specific after training. The following equation is utilized to calculate the classification accuracy of the preference level for each video duration.

(3) $$\begin{array}{r} P=\frac{N_{D}+N_{S}+N_{L}}{N_{T}}\times 100\% \end{array}$$

where *N*_*D*_, *N*_*S*_, and *N*_*L*_ are the numbers of “dislike,” “so-so” and “like” samples after the process of the CNN identification, respectively, and *N*_*T*_ is the number of samples of input data. The classification accuracy is defined as *P* for each trial, and the final classification accuracy is achieved by averaging the results of all trials. There is the same principle to achieve the classification accuracy for pairwise classification results based on different genders.

#### Visualization of Extraction Features

Several extraction and classification methods used in previous studies have not achieved a high classification performance for a large number of samples. Consequently, in this study, to achieve high classification performance, the proposed CNN-based fNIRS structure was used to extract and classify features because of its advantage of automatic feature extraction. In addition to the CNN performance, the visualization approach was utilized to show the results of decoding different preferences and genders. During data processing, it is difficult to visualize high-dimensional data in the classification of preferences. Thus, principal component analysis (PCA) was utilized to reduce the quantity and dimensionality of the data.

In this study, the visualization of extracted features provides insights to analyze the hemodynamic activation and decode the different preferences of consumers. The visualization results are plotted using the first and two principle components of the PCA. The procedure to visualize signal features is shown in the following section.

### Regions of Interest for Preferences

The *t*-value map is a more intuitive approach to show the brain activation according to the fNIRS data. In this study, the *t*-values were computed by using the *robustfit* function available in Matlab in comparison with the expected hemodynamic response. The *t*-value was determined to the human brain cortex activation-related coefficient if the shape of the HbO response is closer to the expected hemodynamic response. The *t*_crt_ value depends on the degrees of freedom (number: *N*−1), if a channel with the computed *t*-value is greater than *t*_*crt*_, the channel is defined as active (Khan et al., 2014). The regions of interest for each subject are investigated through the maps.

## Results

### Behavioral Results

**Figure 6:**
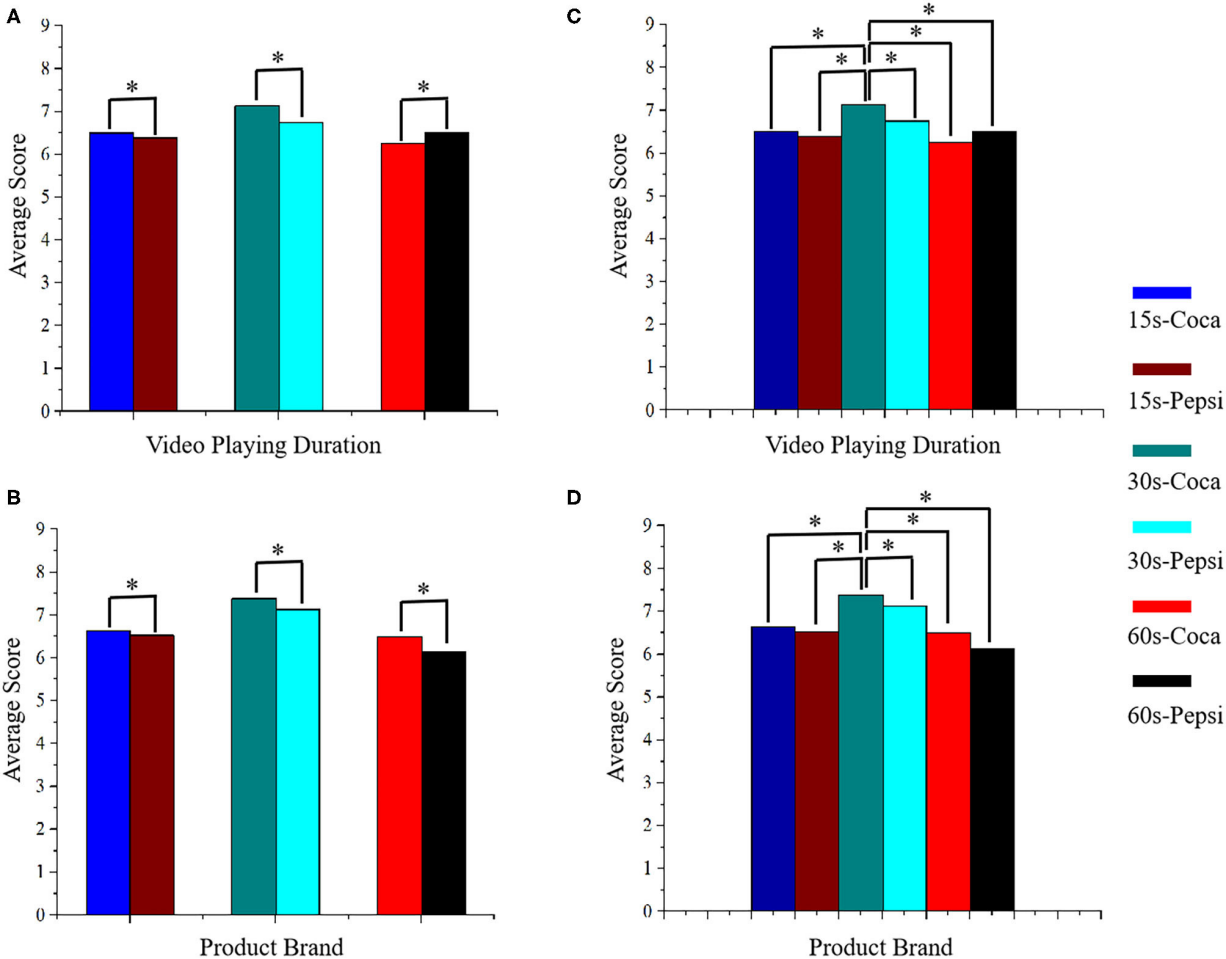
Statistical results of behavioral data: **(A,B)** by independent sample *t*-test, and **(C,D)** by one-way ANOVA. *p < 0.05.

Figure 6 shows behavioral analysis results using two methods called independent sample *t*-test and a one-way ANOVA for different types of videos. The two independent variables, which are video playing duration and product brand, are utilized to analyze the participants' preferences. All the commercial videos are divided into six types based on these variables, including 15 s-Coca, 15 s-Pepsi, 30 s-Coca, 30 s-Pepsi, 60 s-Coca, 60 s-Pepsi, respectively. An independent sample *t-*test was used to analyze the video playing duration and the product brand for the two variables (Figures 6A,C). A one-way ANOVA was utilized to analyze the effects of the six different stimulation types using the two independent variables (Figures 6B,D). The results reported that the video playing duration of 30 s-Coca commercial videos [i.e., mean (M) = 7.13, standard deviation (SD) = 0.835, *p* = 0.043] were significantly higher than those of 30 s-Pepsi commercial videos (i.e., M = 6.75, SD = 1.035, *p* = 0.043). Video playing duration for 60 s-Pepsi commercials videos (M = 6.50, SD = 1.195, *p* = 0.045) was slightly higher than those of 60 s-Coca commercial videos (M = 6.25, SD = 1.035, *p* = 0.045). Also, for the product brand preferences, the 30 s-Coca commercial videos (M = 7.38, SD = 1.061, *p* = 0.035) were greater than the 30 s-Pepsi commercial videos (M = 7.13, SD = 0.641, *p* = 0.035), the 60 s-Coca commercial videos (M = 6.50, SD = 1.069, *p* = 0.037) were significantly higher than the 60 s-Pepsi commercial videos (M = 6.13, SD = 0.835, *p* = 0.037). On the other hand, the video playing duration for the 30 s-Coca cola commercial videos (M = 7.13, SD = 0.835) were significantly higher than the preference scores of the other types: 15 s-Coca videos (M = 6.50, SD = 0.925, *p* = 0.012), 15 s-Pepsi videos (M = 6.38, SD = 0.916, *p* = 0.002), 30 s-Pepsi videos (M = 6.75, SD = 1.035, *p* = 0.000), 60 s-Coca videos (M = 6.25, SD = 1.035, *p* = 0.000), 60 s-Pepsi videos (M = 6.50, SD = 1.195, *p* = 0.002). From the point of the product brand preferences, the 30 s-Coca cola commercial videos (M = 7.38, SD = 1.061) were also significantly higher than the rest of the types: 15 s-Coca videos (M = 6.63, SD = 1.061, *p* = 0.000), 15 s-Pepsi videos (M = 6.51, SD = 0.744, *p* = 0.001), 30 s-Pepsi videos (M = 7.13, SD = 0.641, *p* = 0.005), 60 s-Coca videos (M = 6.50, SD = 1.069, *p* = 0.000), 60 s-Pepsi videos (M = 6.13, SD = 0.835, *p* = 0.012).

### Preferences Classification in Visualization Results

**Figure 7:**
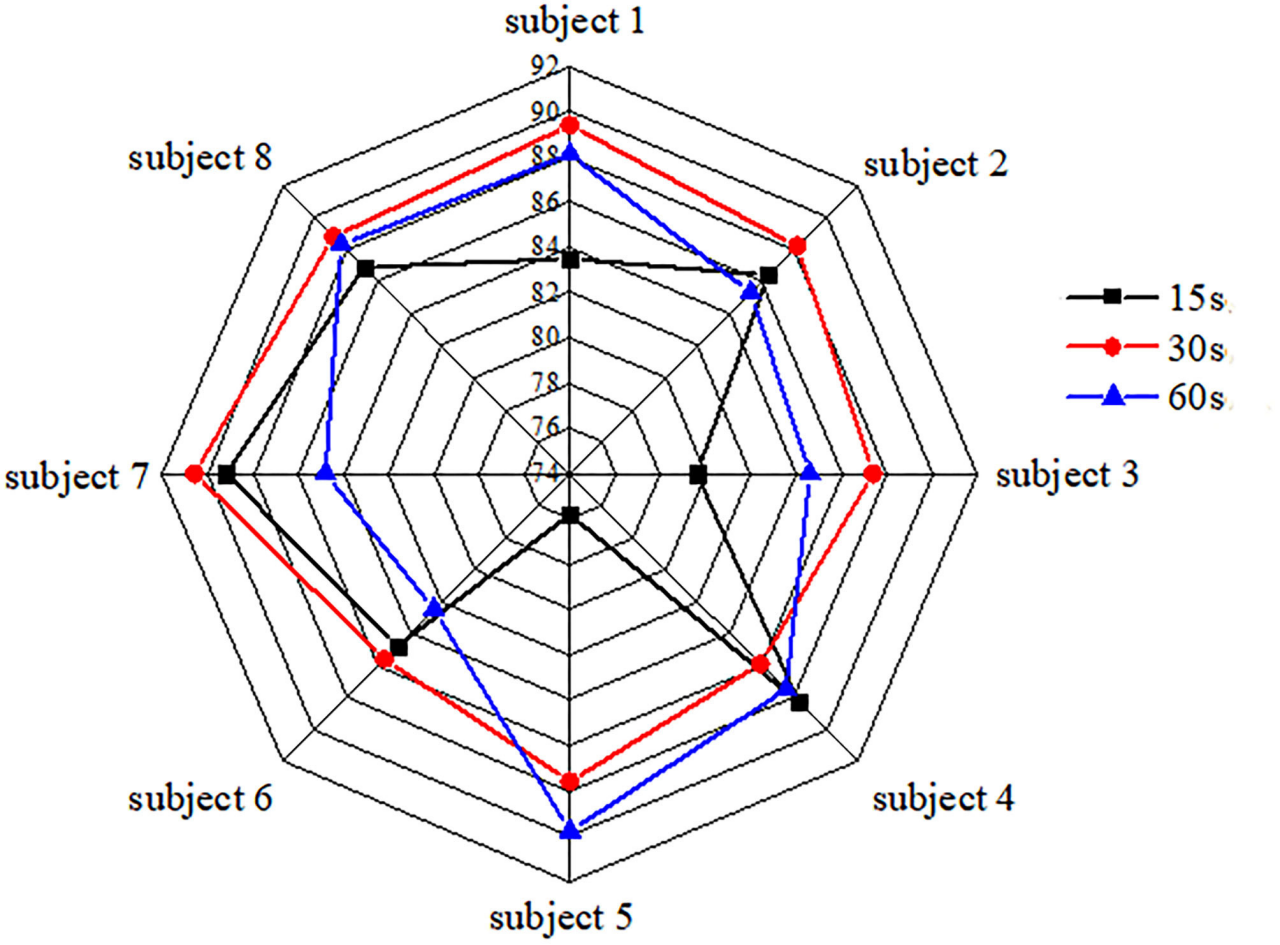
Average classification accuracy of individual subjects for different durations.

**Figure 8:**
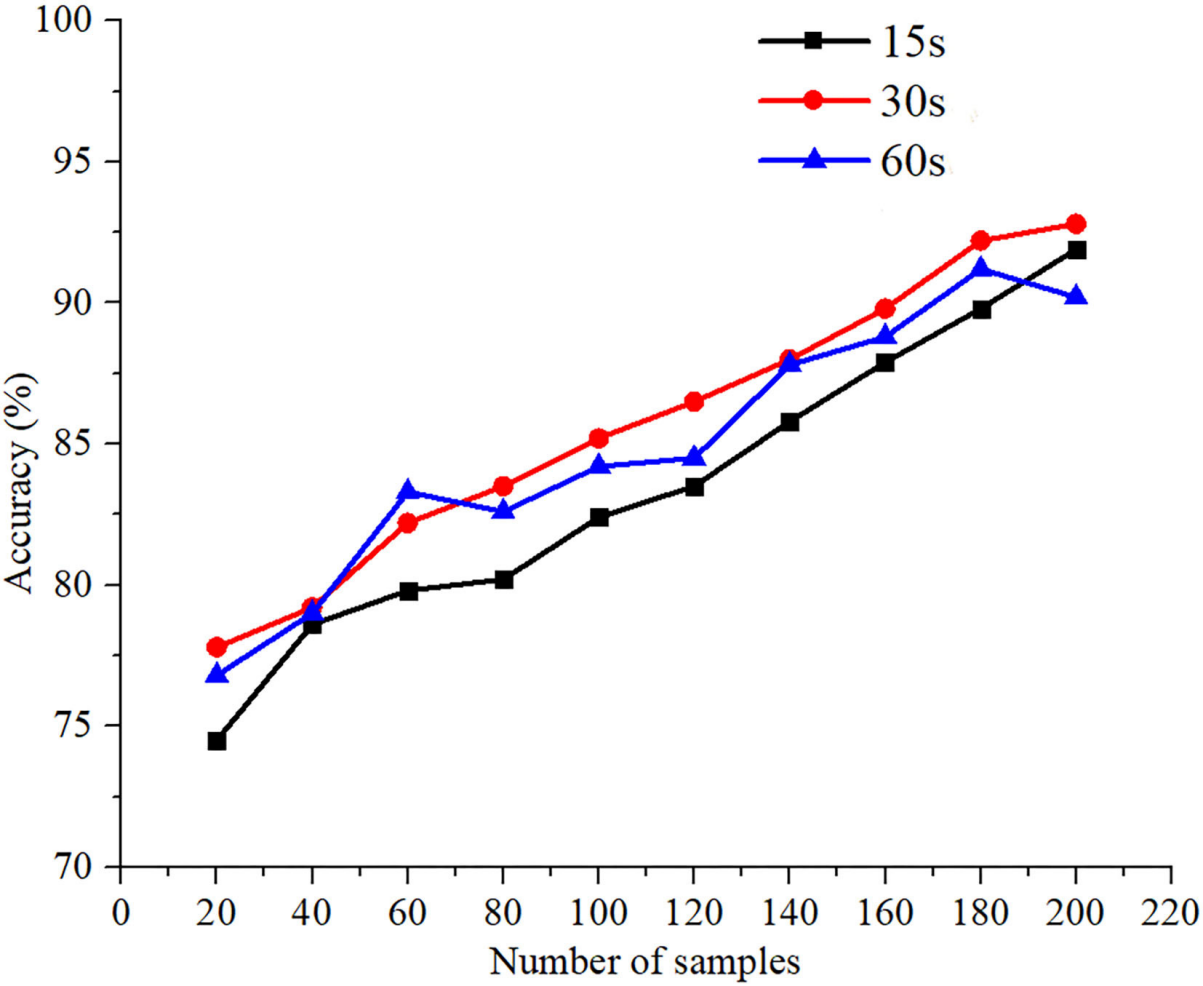
Average classification accuracy of all subjects based on different numbers of samples and different durations.

The classification accuracy of each participant for videos of different durations was used to obtain the overall classification accuracy of each participant by averaging the results across the channels and trials (Figure 7). The average values of the classification accuracy of 15, 30, and 60 s videos are 84.3, 87.9, and 86.4%, respectively. Among them, the classification accuracy of the 30 s video is the highest. From the measurement results of three different preferences of eight participants, participant 7 achieved the highest classification accuracies of 89.2 and 90.6% for the 15 and 30 s videos, respectively, and participant 5 achieved the highest accuracy of 89.8% for the 60 s video. For the 30 s video, the classification accuracies of all participants are over 85% and more explicit to those advertising decisions. Moreover, compared to other durations (Figure 8), the classification accuracy of the 30 s video is the highest when the number of samples is >80, and the CNN achieves an accuracy >83.5 and 90.6% with 80 samples and 200 samples, respectively. As the number of samples is increased for videos of different durations, the increase in classification accuracy decreases gradually and reaches 90%.

Regarding the classification performance (Figures 7, 11 and Table 5) based on gender, it was observed that the preferences of “like” and “dislike” present a superior classification performance for the female and male participants compared to other combinations such as “like” vs. “so–so” and “dislike” vs. “so–so.” Moreover, male participants have targeted preferences for commercial advertising, and the classification performance of “like” and “dislike” was better. Participants 1, 4, 5, and 7 show better visualization results to classify different preference levels after commercial advertising stimulation.

**Table 5:** Preference pairwise-classification results for different genders.

**Gender**		**Duration**	**Like vs. So-So**	**Like vs. Dislike**	**So-So vs. Dislike**
Female	Participant 1	15 s	85.8	87.5	84.6
		30 s	86.7	**88.6**	85.5
		60 s	87.5	87.4	88.6
	Participant 2	15 s	86.6	**88.2**	84.9
		30 s	87.2	87.9	86.6
		60 s	86.1	87.3	84.4
	Participant 3	15 s	**88.2**	87.8	85.7
		30 s	85.5	86.5	85.1
		60 s	82.5	85.2	83.8
	Participant 4	15 s	86.2	**88.7**	85.1
		30 s	87.2	86.9	84.3
		60 s	83.5	87.2	83.6
	Averaged		**86.1**	**87.4**	**85.2**
Male	Participant 5	15 s	87.5	88.5	86.8
		30 s	88.5	89.2	86.5
		60 s	86.8	88.5	89.6
	Participant 6	15 s	84.6	86.9	82.6
		30 s	86.6	**88.6**	84.2
		60 s	85.6	87.6	85.1
	Participant 7	15 s	86.8	90.4	84.2
		30 s	88.6	**90.4**	88.5
		60 s	85.9	88.5	87.8
	Participant 8	15 s	83.5	86.6	80.9
		30 s	83.5	**87.9**	84.6
		60 s	80.5	87.8	83.8
	Averaged		**85.7**	**88.4**	**85.4**

*The bold values indicate the highest classified values for each participant in pairwise classification*.

### Quality of the ROIs in the Prefrontal Cortex

**Figure 9:**
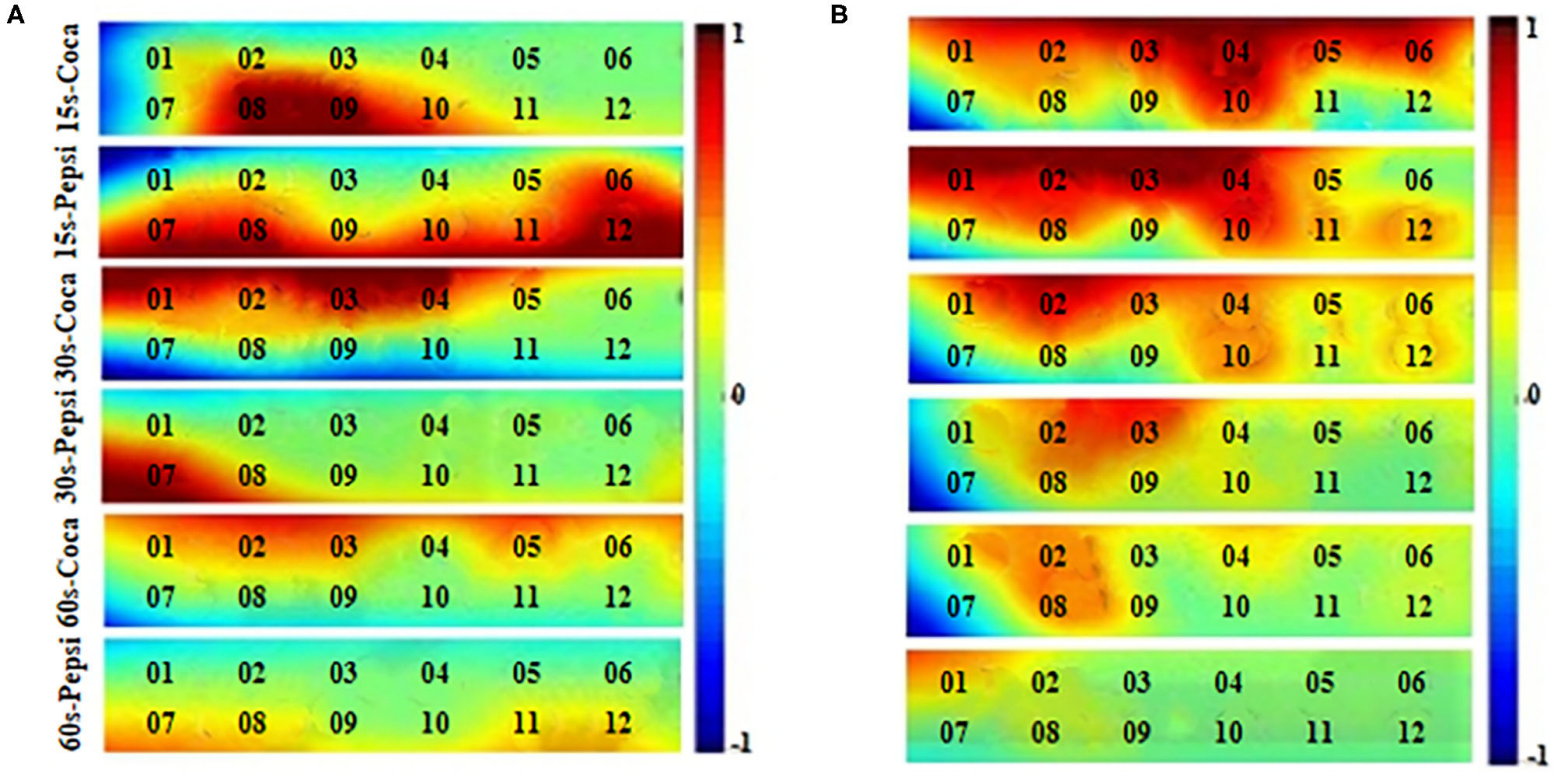
The averaged brain activation maps of six different videos for different genders. **(A)** Averaged female subjects' maps. **(B)** Averaged male subjects' maps.

Figure 9 shows different brain cortex activation maps from eight subjects for commercial videos of 15 s-Coca, 15 s-Pepsi, 30 s-Coca, 30 s-Pepsi, 60 s-Coca, 60 s-Pepsi, which are from the prefrontal cortex. The regions of interest (ROIs) upon different videos were found different. The averaged brain activation maps of different genders over eight subjects were obtained in Figure 9 through the data from the ROI of each trial. Both Figures 9A,B were the averaged female subject maps and the averaged male subject maps based on six different types of videos, respectively. In the female map, the 15 s-Coca, 15 s-Pepsi, and the 30 s-Coca videos showed more activation channels than others. Among them, channels 8, 9, 10, and channels 1, 2, 3, 4 were activated when the 15 s-Coca and 30 s-Coca videos were shown, respectively. Channels 6, 7, 8, 10, 11, and 12 were activated when viewing the 15 s-Coca video. In the male map, the 15 s-Coca, 15 s-Pepsi, and the 30 s-Coca videos also showed more activation than others: Channels 1, 2, 3, 4, 5, 6, and 10 were activated when viewing the 15 s-Coca video, channels 1, 2, 3, 4, 8, and 10 were activated when the 15 s-Pepsi video was shown, channels 1, 2, 4, and 10 were activated when viewing the 30 s-Pepsi video. From the above description, it is considered that the 15 s-Coca, 15 s-Pepsi, 30 s-Coca videos showed more activation channels for both female and male subjects. The results of ROI also suggest that the advertising videos with a shorter duration can generate more region of interest than long-term stimulation. Furthermore, a significant asymmetry of the ROIs was obtained from the prefrontal cortex.

## Discussion

### CNN-Based Neuromarketing Classification Accuracy

**Figure 10:**
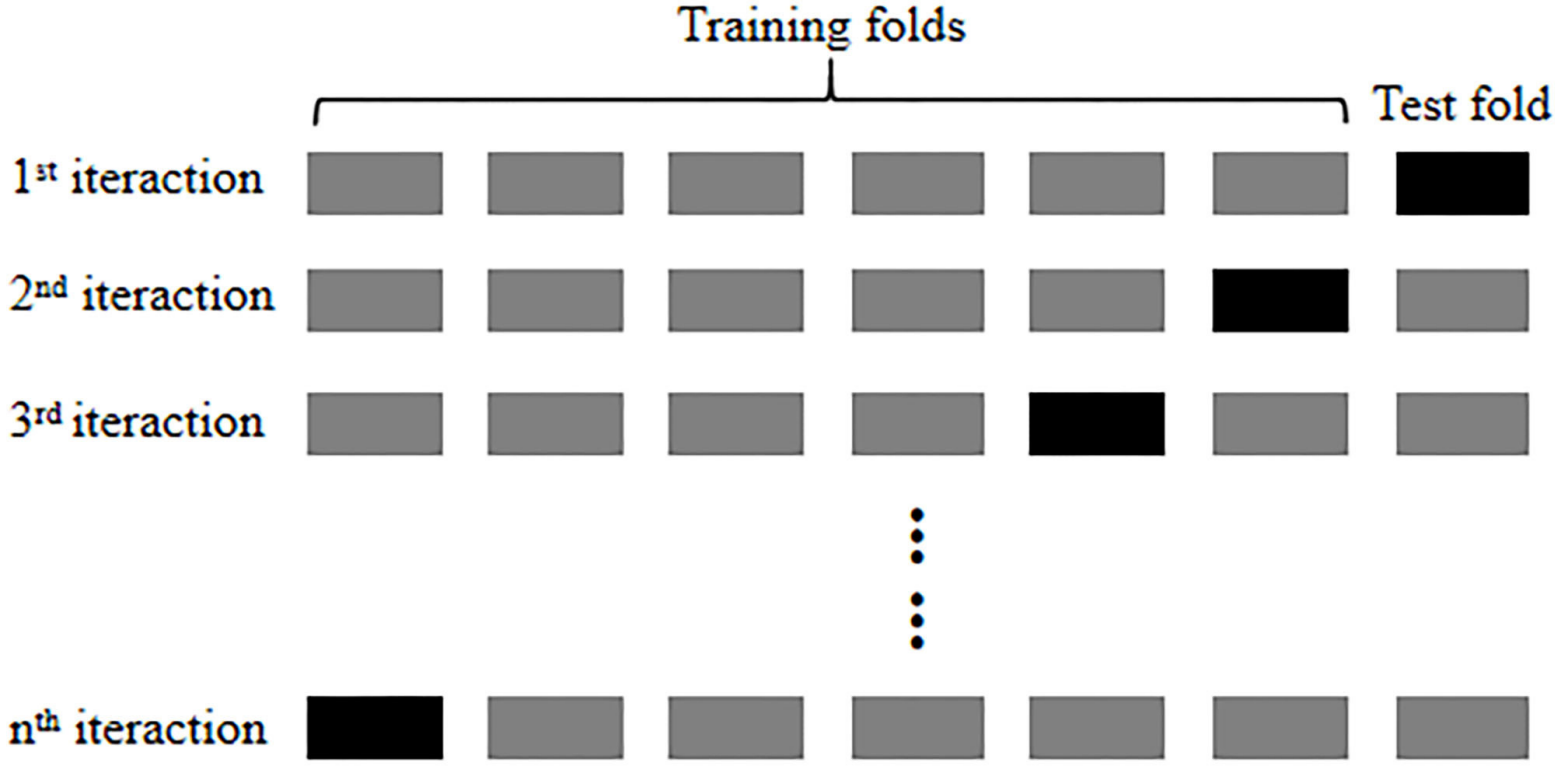
Cross-validation procedure.

To determine the classification accuracy of the CNN and estimate the classification performance of the predictive model (Arlot and Celisse, 2009; Zheng et al., 2019), the 8-fold cross-validation method was utilized in this study to evaluate the predictive model in the point of the classification performance. The first access is to divide the collected data into 8-folds during the process, and an identical amount of the input data are composed of each fold. Then, 1-fold is utilized as a test set to evaluate the model performance, and the rest of the folds are used as training sets to train the proposed model (Figure 10). Finally, a classification procedure is applied to the selected testing and training sets. Each of the 8-folds has performed a critical role in the testing and training processes, and the related accuracies obtained from individual testing sets were averaged to evaluate the model performance. We attempted to discriminate the three cases of preferences, including high-, mid-, and low- activations, and defined them as three different preferences of consumers: “like,” “so–so,” and “dislike,” To achieve a high classification accuracy, the CNN–fNIRS structures were applied to classify common signal features.

In particular, Figure 7 shows the classification accuracy of individual participants, and as expected, the results obtained using the CNN structures show superior classification accuracy compared to conventional methods. The average values of the classification accuracy of the 15, 30, and 60 s videos are 84.3, 87.9, and 86.4%, respectively. Among them, the classification accuracy of the 30 s videos was the highest. From the measurement results of the three different preferences of eight participants, participant 7 achieved the highest accuracies of 89.2 and 90.6% for the 15 and 30 s videos, respectively. Moreover, participant 5 shows the highest accuracy of 89.8% for 60 s-video. To achieve a high classification performance for videos with different durations, the automatic learning ability of the CNN to process an input dataset was crucial to achieving a superior classifier, and the weight values of the convolutional filters were updated using the inherent convolutional patterns.

The size of the training dataset as a critical element affected the learning performance, and this is especially true for CNNs and other artificial algorithms. Furthermore, to examine the relationship between the size of the dataset and classification accuracy, the average values of the classification accuracy of all participants were obtained. The 8-fold cross-validation method was utilized to evaluate the CNN classification performance. For all classification accuracies at different durations, it was observed that the classification performance increased with the number of samples in the data set. Figure 8 shows the classification accuracy of CNN for different numbers of samples. Moreover, compared to other durations, the classification accuracy of the 30 s video is the highest when the number of samples is >80. Furthermore, CNN achieves >83.5 and 90.6% accuracy for 80 samples and 200 samples, respectively. With the increase in the number of samples, the classification performance of the preferences also improves. Thus, to further classify the different preferences and decision-making of consumers, more participants and the number of samples should be considered.

### Visualization of Different Preferences Levels Using CNN

**Figure 11:**
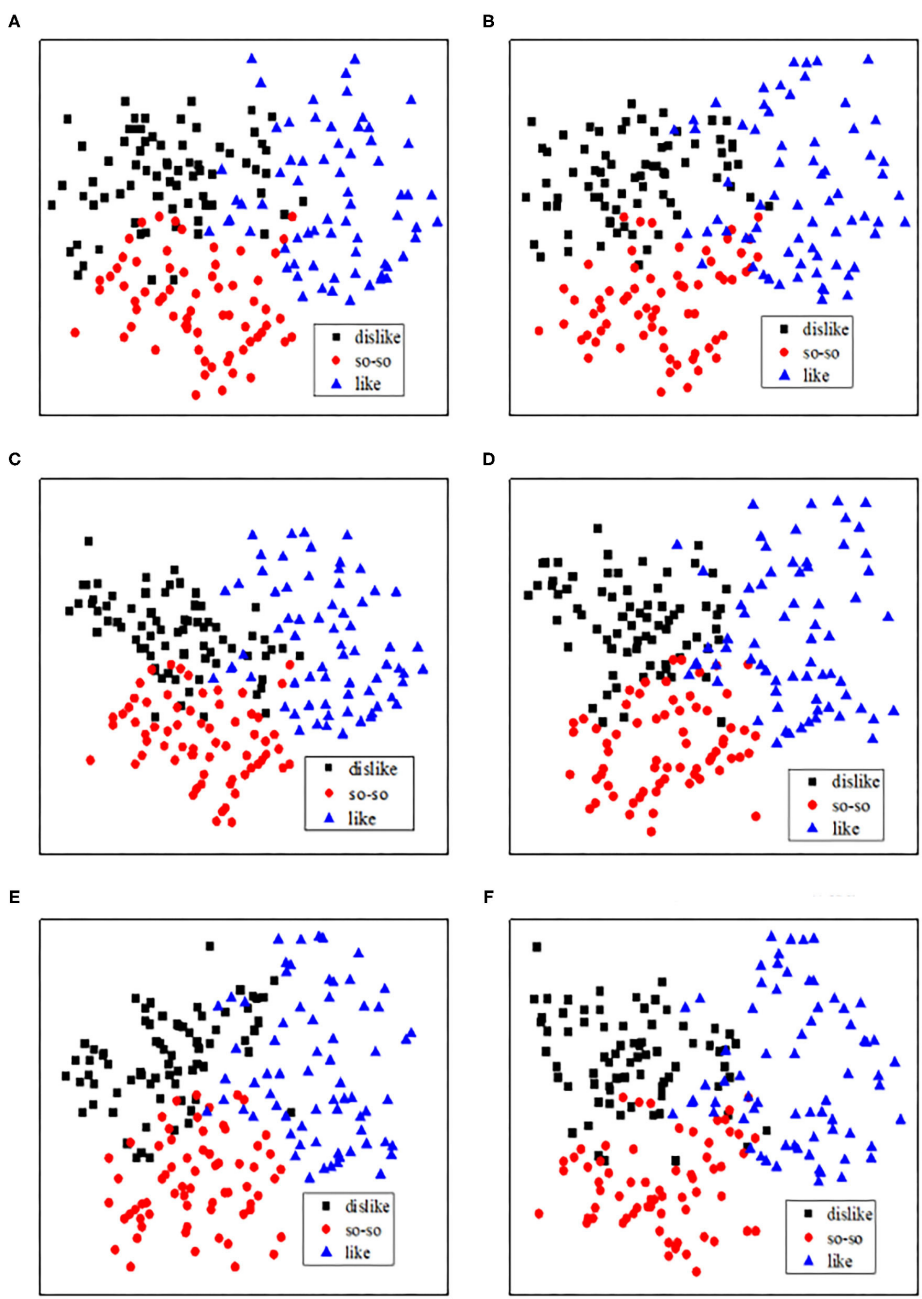
Preference classifications: The visualization of the hemodynamic response signals using CNN for different genders and durations. **(A)** Female (participant 4): 15 s-advertising. **(B)** Male (participant 7): 15 s-advertising. **(C)** Female (participant 1): 30 s-advertising. **(D)** Male (participant 7): 30 s-advertising. **(E)** Female (participant 1): 60 s-advertising. **(F)** Male (participant 5): 60 s-advertising.

To decode the different preference levels of consumers using CNN-based fNIRS and to better understand the decoding results and feature extraction performance, we visualized three cases of high-, mid-, and low-activations (defined as different preference levels: “like,” “so-so,” and “dislike,” respectively). In particular, three cases were visualized using convolutional processing. Figure 11 shows the results of the PCA in the first and second principles. The results of female participants 1 and 4 and those of male participants 5 and 7 show that the extracted features using convolutional filters were better discriminated at different activation levels for different commercial advertisements, and the results are compared based on gender.

The classification performances (Figures 7, 11), indicated that, in the case of male participants, the preferences of “like” and “dislike” present superior classification performance compared to other combinations such as “like” vs. “so-so” and “dislike” vs. “so–so.” In particular, it is easy to classify wide-range hemodynamic responses using CNN-based fNIRS. In contrast, in the case of female participants, the visualization of different consumer preferences shows a good classification performance. Compared to female participants, there are specific differences in the preference decision-making levels of male participants. Among them, male participants have more targeted preferences for commercial advertising, and the classification performances of “like” and “dislike” were better.

To decode the different preference levels of consumers in neuromarketing, a novel and automatic method, which includes a CNN algorithm and decoding process, is utilized to explore consumer behavior and intention. Compared to other general extraction and classification feature methods, the CNN-based fNIRS method shows superior classification and visualization performance in this study. In future work, other CNN structures and optimized decoding processes should be considered to improve classification accuracy.

### Prefronal Cortex Activation in the Preferences

The prefrontal cortex (PFC) area is a crucial part of the whole brain cortex, and it has been implicated in decision-making, moderating social behavior, planning complex cognitive behavior, etc. In this work, the prefrontal cortex for activation maps (Figure 9) had presented the activated channels when the participants were stimulated with different types of commercial videos. From the viewpoint of the activated maps, compared to the PFC's right area, the PFC's left and center regions had higher cortex activation when the 15 s-Coca, 30 s-Coca, and 30 s-Pepsi videos were played before female participants. Otherwise, the other types of videos have shown mostly similar activation between the compared PFC area. On the other hand, from the insight of the male participants' activation map, the 15 s-Pepsi, 30 s-Coca, and 30 s-Pepsi videos are significant higher activation in the left and center region of the PFC compared to the right region. Also, the 60 s-Coca and 60 s-Pepsi videos are slightly higher activation except for the 15 s-Coca video. It is concluded that the PFC's left and central regions play a critical role in the decision-making, preference-related behavior, and positive behavioral cognition when attractive commercial videos are shown.

## Limitation and Future Prospects

In this study, the number of participants (eight participants) for the training and testing process of the CNN structures was less than the real number of classification preference levels. With respect to consumer behavior cognition, many impact factors cause changes in consumer decision-making, and the experiment was designed and developed without any changes in the surroundings. To solve these problems, a large number of participants should be involved in this experiment to optimize the deep neural network model and further enhance the stability and universality of this model. Through the continuous optimization of the decoding model, high classification accuracy can be achieved for different consumers in any specific commercial advertisement, and consequently, the corporate and sales marketing can obtain more accurate information about product involvement.

## Conclusion

This study demonstrated fNIRS-based classification of high-, mid-, and low-activation using a CNN as the classifier in the field of neuromarketing, and compared the classification performance of the visualization results of participants in an experiment. The participants were instructed to focus on commercial advertisements of different durations (15, 30, 60 s), as displayed on a computer monitor. The high-, mid-, and low-activation, which were referred to as different preference levels: “like,” “so-so,” and “dislike,” were classified using CNN along with various features, such as mean, peak, slope, variance, kurtosis, and skewness. From the measurement results of three different preference levels of eight participants, a superior classification accuracy of 87.9% for the 30 s advertisement video was observed compared to videos of other durations. The classification performance of participant 7 showed the highest accuracies of 89.2 and 90.6% for the 15 and 30 s videos, respectively, and participant 5 achieved the highest classification accuracy of 89.8% for the 60 s video. From the results of the classification visualization, the male participants were observed to have targeted preferences for commercial advertising compared to female participants, and the classification performances of “like” and “dislike” were better. The results of the CNN-based fNIRS, which present a good classification performance, indicate the applicability of BCIs in neuromarketing, which can be used in the practical development of the BCI systems.

Because the classification performance is a critical factor in decoding the preferences of the consumers, and the superiority of CNN as a superior classifier over other conventional methods has been reported in other publications, we plan to optimize the performance of the CNN-based neuromarketing systems by applying various deep neural networks, and develop novel approaches with respect to hybrid imaging modalities such as combining electroencephalography with fNIRS.

## Data Availability Statement

The datasets presented in this article are not readily available because the datasets generated for this study are available on request to the corresponding author. Requests to access the datasets should be directed to kshong@pusan.ac.kr (Keum-Shik Hong).

## Ethics Statement

The studies involving human participants were reviewed and approved by Pusan National University Institutional Review Board. The patients/participants provided their written informed consent to participate in this study.

## Author Contributions

KQ carried out the data processing and wrote the first draft of the manuscript. RH participated in collecting experimental data. K-SH suggested the theoretical aspects of the current study, corrected the manuscript, and supervised the entire process leading to the manuscript generation. All authors contributed to the article and approved the submitted version.

## Conflict of Interest

The authors declare that the research was conducted in the absence of any commercial or financial relationships that could be construed as a potential conflict of interest.
